# Some Remarks on Hernia

**Published:** 1908-12-05

**Authors:** 


### SURGERY.

### SOME REMARKS ON HERNIA.

### VI. THE OPERATIVE TREATMENT OF A STRANGULATED HERNIA (*continued*).

We have described in a previous article the steps which must be taken in dealing with a strangulated hernia, with the single exception of the case in which the gut in the hernia is obviously incapable of surviving. But it will be asked, How can this be known? Intestine which is completely gangrenous is black or grey in colour, and has lost the glistening appearance which is characteristic of normal gut. It does not bleed when it is pricked, and often has a peculiar sensation of "crackling" when handled: this is due to the fact that there is nearly always surgical emphysema. Further, it emits a very unpleasant odour. Any of these signs is by itself sufficient evidence that the affected bowel will surely necrose. What, then, should the surgeon do in face of such a contingency? The problem may be a most difficult one, since every moment is of value, and he must decide immediately which of the alternative methods at his disposal he will adopt.

One law may be laid down—namely, that so long as the patient's symptoms are relieved, the less done in the way of a set operation the better. Treves, in his "System of Surgery," advocated an incision into the intestine *to relieve the obstruction*. This is the first essential. Further, he laid great stress on doing this merely, and not dividing the constricting agent at the time, his argument being that to do this incurs the risk of separating adhesions which have formed between the damaged intestine and the neck of the sac, and thus allowing some portion of the intestinal contents, which are violently toxic to the peritoneum, to enter the general cavity; and he stated, moreover, that in his experience the constriction rarely, if ever, prevented a flow of fæces from the intestine. If it did, he advised the insertion of a pair of scissors into the lumen of the bowel and stretching the orifice by opening the blades when in this position. These arguments are doubtless as sound to-day as when they were written; but a great change has come over our ideas as to the powers of the peritoneum during recent years; and, in the light of our present knowledge, it appears that too much stress was laid upon this point. It must, indeed, be quite rare to get a general infection of the peritoneal cavity after division of the constricting agent. It would be far more likely, if the constriction was divided and the gut pulled down, that fresh adhesions would quickly form and fix the intestine down into its new position. In any case, even if it were infected from the gut, the infection would probably be a local one if there was free drainage of the wound.

The step that we advise is therefore to cut the constriction and pull down the coil of gut. The gangrenous portion is then boldly cut away, and the two cut ends fixed in the wound, with a suture through the serous coat, if necessary. A Paul's tube should then be tied into *each* end, the wound left open, and dressings applied. It is important to tie a Paul's tube into each end and not into one only, because it is difficult to know which is the proximal and which the distal end in the altered state of the parts. A fæcal fistula is thus established which, if it acts properly, will relieve the patient of all his symptoms; and the operation has the great advantage that it can be performed in a few minutes—a matter of great importance in these cases, since the patient is already exhausted. Later on, steps will have to be taken to close the fæcal fistula.

In dealing with any case of intestinal obstruction, which either is from the beginning acute or which has become acute, it is a sound rule to do as little as possible in the acute stage, provided that the symptoms are relieved. The cause can be dealt with later. There are, of course, obvious exceptions to this. An early intussusception which can be reduced at the outset is a case in point. But it is our opinion that the rule holds good in a gangrenous hernia. Some authorities maintain, however, that the patient has a better chance of ultimate recovery if the gangrenous intestine is immediately resected and an end-to-end anastomosis performed at the same time.

This can be done in one of two ways. In the first the original wound is enlarged, the constriction divided, the intestine pulled down, and the gangrenous portion boldly cut away. The ends are then united in the wound either by simple suture, if feasible, or by means of a Murphy's button. This has the advantage of reducing the time of the operation, but the material disadvantage that the hernial opening in the abdominal wall must be greatly enlarged in order to admit the button when the intestine is replaced; and if the hernia be femoral, it necessitates cutting Poupart's ligament, which is a grave step, since the parietes are much weakened thereby.

An alternative operation has therefore been proposed. In this, as soon as the state of affairs is recognised, the abdomen is opened by a vertical incision in the lower half of the corresponding rectus abdominis muscle, and the two coils, the afferent and efferent, leading respectively to and from the inner aspect of the hernial orifice are sought for. The intestine is divided in two places, the ends belonging to the herniated intestine being ligatured, while the other two are united by an end-to-end anastomosis. The gangrenous portion which is thus completely freed from the intestinal canal is pulled down, cut away from its mesenteric attachment, and removed: the rent in the mesentery is remedied *secundum artem* through the abdominal wound. This is then sewn up, and a large drainage tube put into the first wound, which is only partially closed.

We do not personally regard either of these proceedings with favour, for the following reasons: The patient's general condition is commonly such that a long and complicated operation is not justifiable; and, even if he survives the immediate effects of the operation, the results of end-to-end anastomosis in intestine which has been in a state of acute obstruction, are, in our experience, uniformly unsatisfactory, since in many cases the union leaks and fatal peritonitis is set up.

